# Phospholipase C is a novel regulator at the early stages of microspore embryogenesis in *Nicotiana tabacum*

**DOI:** 10.1080/15592324.2022.2094618

**Published:** 2022-07-04

**Authors:** Pan Luo, Aixi Jiang, Yi Zhou, Mingchun Yang, Xiaotong Zhou, Yong Yang, Jun Yu, Xingchun Tang

**Affiliations:** a State Key Laboratory of Biocatalysis and Enzyme Engineering, School of Life Sciences, Hubei University, Wuhan,Hubei China; b Tobacco Research Institute of Hubei ProvinceWuhan, Hubei, China

**Keywords:** Phospholipase C, microspore embryogenesis, Nicotiana tabacum

## Abstract

Microspore transfers the developmental fate into embryogenesis in vitro regulated by determinant factors of stress-induced. However, the key regulators of microspore embryogenesis (ME) are still largely undiscovered to reveal the mechanism of cell fate transition. Here, we report that Phospholipase C (PLC) is involved at the early stages of ME in *Nicotiana tabacum. NtPLC2/3/4* are expressed at the initial stages of ME. The expression levels of *NtPLC2/3* are transient activated after 3 days in culture, while the expression level of *NtPLC4* maintains relatively stable. Inhibition of PLCs induces the decrease in *NtPLC2/3/4* expression level and decline of ME yield. We confirm that lipids in microspore are degraded and then re-accumulate at first embryonic division stage. Inhibition of PLCs suppresses the lipids metabolism at the early stages of ME. Thus, we propose that PLCs-mediated lipid metabolism is a novel regulator at the early stages of ME.

## Introduction

Microspores can be reprogrammed by stress treatment in vitro to shift from gametophytic development toward a sporophytic pathway, a process known as androgenesis,^1^ or microspore embryogenesis (ME). It has been extensively exploited in plant-breeding programs to increase the speed and efficiency with which homozygous lines can be obtained.^1^ Due to the advantages of convenient access to materials and easy operation, ME has been exploited for planting breeding^2^ and as a model of studying cell fate transition^3^ in recent decades. Usually, microspores required to induce embryogenic growth are the application of stress treatment like osmotic, temperature, or nutrient stress, either alone or in combination.^4^ Although several regulators of ME have been found in previous researches, such as chromatin regulation,^5^ small RNA,^6^ calcium signaling,^7^ and hormones,^8^ the mechanism underlying the decision in microspore cell fate is still largely unknown, and the signaling pathways activated by stress-induced need to be further explored.

Phospholipids are important components of cytoplasmic membranes, and phospholipases are responsible for the hydrolysis of phospholipids. Based on the target position of hydrolysis of glycerophospholipid, plant phospholipases are classified into phospholipase A (PLA), phospholipase C (PLC), and phospholipase D (PLD).^9^ Five phosphoinositide-specific phospholipase C (PI-PLC) isoforms, β, γ, δ, ε, and ζ, have been identified in mammals, and the structure of plant PI-PLCs is highly similar to mammalian PI-PLC-ζ isoform.^9^ PLC pathway is a mainly synthesis pathway of phosphatidic acid in plants, which acts as a lipid second messenger and is involved in a wide range of defense responses.^10^ These studies report that transcriptional activation of the *PI-PLC* gene family is important for adapting plants to stress environments.^10^

To date, four *PLC* genes, *NtPLC1, NtPLC2, NtPLC3*, and *NtPLC4*, have been identified in the tobacco.^11^ The tobacco ME is a typical stress-induced cell fate transition. Therefore, it is worth to study whether PLC plays a role at the early stages of ME. Here, we report PLC as a novel regulator in ME and provide evidences for that *NtPLC2/3/4* are involved at the early stages of ME and inhibition of PLCs could suppress ME.

## Results and Discussion

### *NtPLCs* were expressed at the early stages of ME

**Figure 1.**
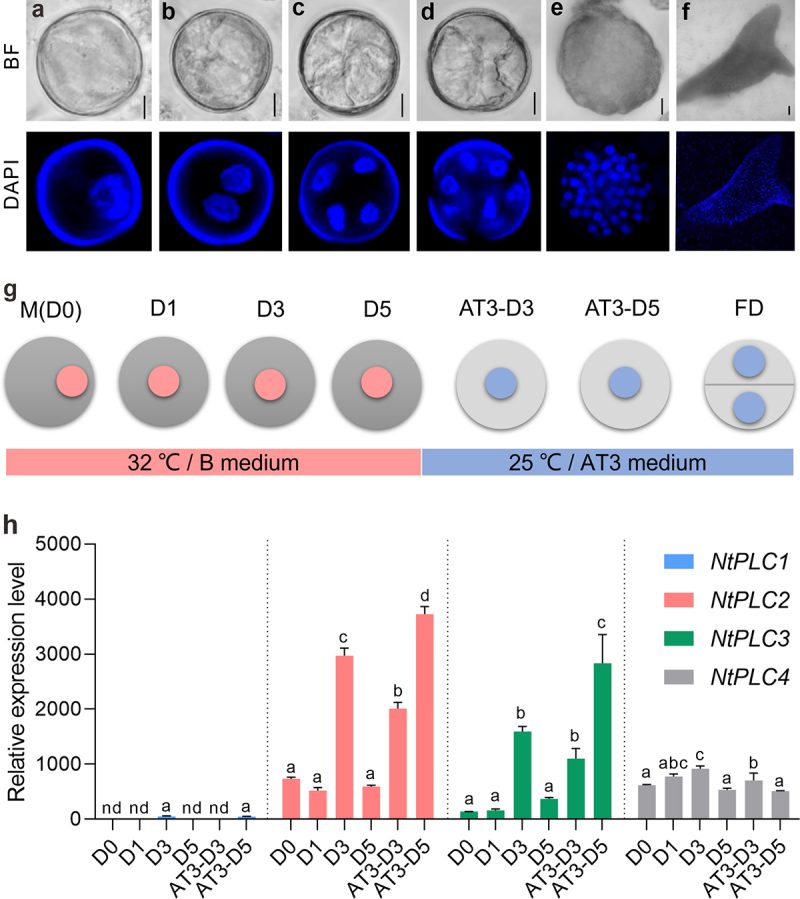
*NtPLCs* were expressed at the early stages of microspore embryogenesis. (a) Late-mononuclear stage. (b) First equally division. (c) Second equally division. (d-e) Globular embryo. (f) Cotyledon embryo. (g) The initial stages of ME in tobacco. D0, microspore; D1, D3, D5 (1, 3, and 5 days in B medium); AT3-D3, AT3-D5 (3 and 5 days in AT3 medium); FD, First division. (h) Relative expression levels of *NtPLCs* at the early stages of ME. nd, no detected. Data are the means ± SD (standard deviations). Letters indicate statistical groups deduced from One-Way ANOVA analysis (Tukey test, *P* < 0.05) performed on the data. Bars: 10 μm in (a-e) and 100 μm in (f).

The microspore population in the late unicellular stage which with big vacuoles and peripherally located nucleus were used for ME culturing (Figure 1a). The initial stage of tobacco ME is induced by heat shock in combination with starvation treatment. Cultured in B-starvation medium for 5 days at 32°C, microspores were transferred into a sugar-containing AT3 medium at 25°C in the dark for embryogenesis. The first sporophytic division (FD) of starvation and heat shock-induced embryogenic microspores were observed after 5–7 days of culture in AT3 medium (Figure 1b). The pattern of subsequent division was similar to that of the zygotic embryo. Rapid cell divisions continued in vitro for 5–6 weeks and resulted in the formation of heart-shaped embryos (Figure 1c-f).

The cell fate transition process is divided into several developmental stages (Figure 1g), including unicellular microspores (D0), 1, 3, 5 day(s) in B medium (D1, D3, and D5), and 3 and 5 days in AT3 medium (AT3-D3 and AT3-D5). qRT-PCR approach was applied to investigate the expression pattern of each *NtPLC* at different stages of early ME. The dynamic expression changes of *NtPLCs* showed different trends. *NtPLC1* was hardly detected in the all stages, indicating that it was not involved at the early stages of ME (Figure 1h). And the expression of *NtPLC4* showed relatively stable during the culture (Figure 1h). In contrast, the expression of *NtPLC2* and *NtPLC3* significantly increased at D3 stage, then decreased at D5 stage. After transferred into AT3 medium, the expression of *NtPLC2* and *NtPLC3* gradually increased again at AT3-D3 and AT3-D5 stages (Figure 1h). Taken together, these data imply the involvement of *NtPLC2*/*3*/*4* at the early stages of ME in tobacco.

### Sequence analysis of NtPLCs

To analyze the evolutionary relationships of PLC proteins, the PLC protein sequences from tobacco and common organisms that have been reported to undergo ME^2^ were aligned and used for a phylogenetic analysis. The PLCs from tobacco, rice, wheat, rape, barley, and *Arabidopsis* (Table S1) were used for the construction of the phylogenetic tree. According to the evolutionary relationships, these PLCs could be grouped as four distinct clusters: two dicotyledon groups and two monocotyledon groups (Figure S1). In terms of evolutionary relationship, the four NtPLCs were more closely related to BnaPI-PLC7Cnn-1, BnaPI-PLC7A9, BnaPI-PLC7Cnn-2, BnaPI-PLC2C1, BnaPI-PLC2C5, BnaPI-PLC2A5, BnaPI-PLC6C4, BnaPI-PLC6C3, BnaPI-PLC6A3, AtPI-PLC2, AtPI-PLC6, and AtPI-PLC7. Thus, we speculate that these BnPI-PLCs may be involved in ME of *Brassica napus*.

Furthermore, the conserved regions of the cluster containing NtPLCs were carried out (Figure S2). Three conserved regions distributed on the front, middle, and rear segments of the protein sequence, and they were X domain of catalytic part (PI -PLC X-box), Y domain of catalytic part (PI-PLC Y-box), and C2 domain in PI-PLC, respectively. In these conserved domains, some specific amino acid sites of NtPLCs were different from other PI-PLC protein in the clade, and these amino acid sites might be unique in tobacco (Figure S2).

During the early stages of ME, *NtPLC2* and *NtPLC3* exhibited significant transcriptional levels increase at D3 stage, and then they reduced at D5 stage. However, expression of *NtPLC4* was tended to stabilize during the whole stages. In addition, NtPLC2 and NtPLC3 showed a higher similarity of protein sequence than NtPLC4 (Figure S2). It implies that *NtPLC2* and *NtPLC3* may have different functions from *NtPLC4* during the early stages of ME.

### Suppressing PLC activity induce decline of ME

**Figure 2.**
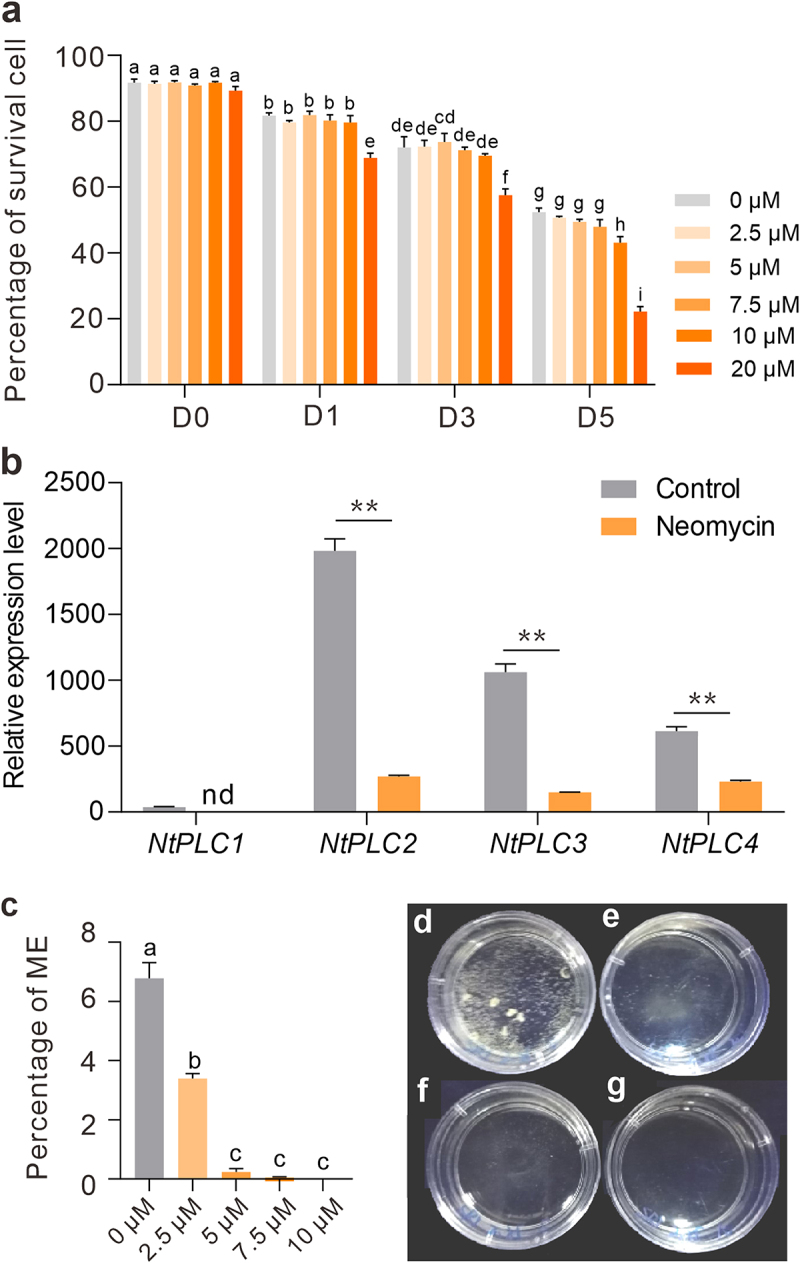
Effects of PLC inhibition on microspore survival, transcription of *NtPLCs* and embryogenesis induction efficiency. (a) Statistics of the percentage of survival cells with 0–20 μM of neomycin treatment at D0 – D5 stages, respectively. (b) Relative expression levels of *NtPLCs* with 5 μM of neomycin treatment for 3 days. (Student’s *t*-test; **P < 0.01). nd, no detected. (c) Statistics of microspore embryogenesis initiation with neomycin treatment at AT3-D6 stage. (d-g) Microspore-derived embryos after 20 d in culture with 2.5 μM (d), 5 μM (e), 7.5 μM (f), and 10 μM (g) of neomycin, respectively. Data are the means ± SD. Letters indicate statistical groups deduced from One-Way ANOVA analysis (Tukey test, *P* < 0.05) performed on the data.

To investigate the role of PLCs in the ME, studying the effects of the PCL-inhibitor neomycin was carried out. Neomycin is a positively charged aminoglycoside binding to PtdInsP_2_ and is used as an inhibitor of PLC.^12^ To eliminate the negative effects on cell vivacity, 2.5 μM, 5 μM, 7.5 μM, 10 μM, and 20 μM of neomycin were added to B medium and tested cell survival with FDA staining, respectively (Figure 2a). In 2.5 μM, 5 μM, and 7.5 μM of neomycin treatment groups, there were no significant differences (*P* > 0.05, One-Way ANOVA) in cell survival, respectively. Decreased cell survival occurred with over 10 μM of neomycin treatment at D5 stage (Figure 2a). Next, the effects of PLC inhibition on the expression level of *NtPLCs* were also evaluated. At D3 stage, qRT-PCR analysis showed that the expression level of *NtPLC2/3/4* in cultures treated with neomycin was significantly lower than control (Figure 2b). These results clarify that inhibition of PLCs can induce decrease of *NtPLC2/3/4* expression at the early stages of ME.

Furthermore, we examined the effects of PLC inhibition on ME initiation. After 6 days in AT3 medium, the number of proembryos was determined as an indicator of initiation of ME. In control, 6.78% microspores could be undergoing embryogenesis (Figure 2c). However, in 2.5 μM of neomycin treatment group, the production was dropped to 3.39%, and proembryos were barely detectable in over 5 μM of neomycin treatment groups (Figure 2c-g). The results demonstrate a significant decrease in ME treated with the PLC inhibitor.

In summary, these data indicate that inhibition of PLCs decline embryogenesis initiation during ME. However, it is still far to know PLCs-mediated signaling in ME to date. In previous studies, it provides some clues about PLCs-mediated signaling in stress responses and plant innate immunity, including protein phosphorylation, alteration in the composition of membrane phospholipids, increase in cytosolic Ca^2+^, NO, and ROS generation, the activities of transporters, protein kinases and transcription factors, and pH in cytoplasm.^13^ Therefore, it is worth to test these downstream effects of PLCs signaling pathway during ME in the near future.

### PLCs play a role in ME via lipid degradation

**Figure 3.**
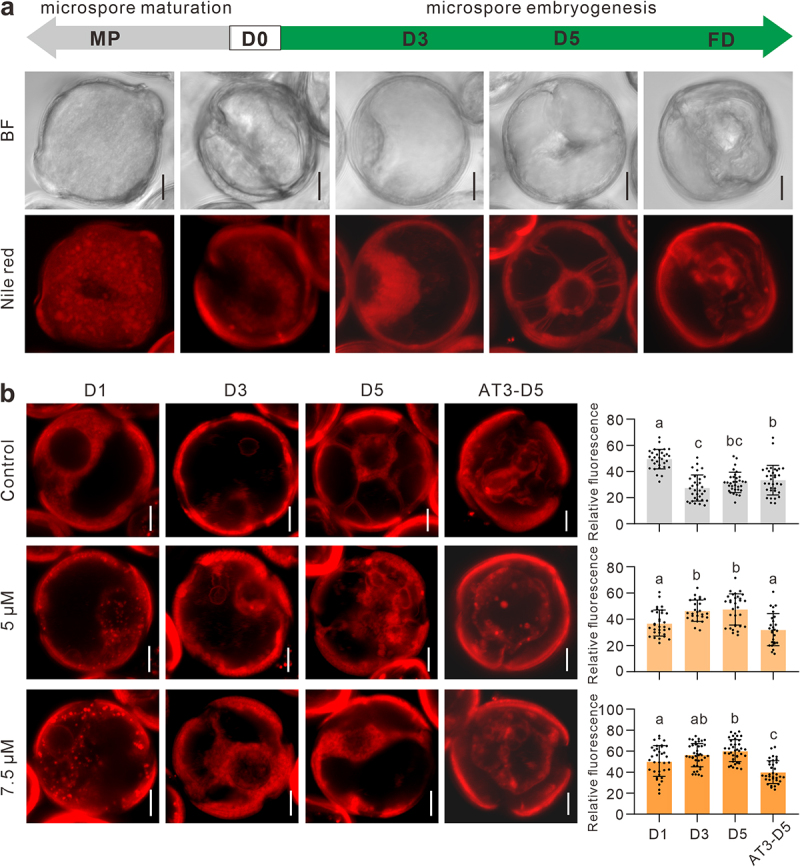
PLC inhibition suppresses lipid metabolism in ME. (a) Nile red staining of microspores in two different developmental pathways. A star-like morphology has been shown in D5. MP, mature pollen; D0, microspore; D3, D5 (3 and 5 days in B medium); AT3-D3, AT3-D5 (3 and 5 days in AT3 medium); FD, first division. (b) The comparative morphology of microspores with Nile red staining. Compared with the control, the B medium of the treatment group contained 5 μM and 7.5 μM of neomycin, respectively. Relative fluorescence intensity of lipid droplets (n = 29–40). Data are the means ± SD. Letters indicate statistical groups deduced from One-Way ANOVA analysis (Tukey test, *P* < 0.05) performed on the data. Bars: 10 μm.

Phospholipases mediate the membrane lipid remodeling as they catalyze the initial step of phospholipid breakdown and generate multiple lipid derived second messengers.^14^ PLCs are involved in lipids signaling and hydrolyze membrane-associated phospholipid, phosphatidylinositol-4,5-bisphosphate to produce two second messengers, inositol-1,4,5-trisphosphate and diacylglycerol.^15^ In ME, the cell fate transition occurs before the cell division, accompanying with a series of cellular component remodeling processes.^16^ We observed lipids in both developmental pathway of gametogenesis and ME. In gametophytic pathway, mature pollen showed a clear and consistent increase as comparing to microspore using Nile red staining (Figure 3a). In contrary, in ME pathway, lipid metabolism presented a dynamic process of first degradation and then re-accumulation, accompanied by typical cytological changes (Figure 3a). In B medium, lipids in the microspores were gradually degraded (Figure 3a). At D5 stage, there were almost no obvious lipid droplets remaining in the cells and showing a star-like morphology formed by the typical cytoplasmic inner membrane remained, and it was considered as the first sign of embryogenic induction. Lipid droplets were observed to re-accumulate within the microspores in AT3 medium (Figure 3a).

Next, to examine the effect of PLC inhibition on lipid metabolism at the early stages of ME, we treated microspores with 5 μM and 7.5 μM of neomycin, respectively. At D1 – D5 stages, neomycin treatment clearly reduced the lipid metabolism by Nile red staining (Figure 3b). In the treatment groups, the accumulation of lipid droplets at D1 stage increased compared with control, and this phenotype aggravated with the increase in the neomycin concentration (Figure 3b). In B medium, lipid degradation was delayed, and the residual amount of lipid droplets was also higher after transfer into AT3 medium (Figure 3b). During the culture, star-like microspores were not observed in neomycin treatment groups (Figure 3b). Therefore, we infer that PLCs inhibition reduces lipid metabolism, which may make a negative effect on the initiation of ME. Remarkably, lipids were abundant when the microspore is developed into a mature pollen, however, they were required to be metabolized when microspore turned to embryogenic development pathway (Figure 3a). Thus, the mechanism of PLCs-mediated lipids metabolism in ME will need further work to reveal.

## Conclusion

In conclusion, *NtPLC2/3/4* could be expressed at early stages of ME, and inhibition of PLCs could reduce ME productivity and lead to lipid accumulation in microspore. We preliminarily studied the expression and role of *PLCs* in ME, and found PLC as a novel regulatory factor of ME in tobacco. These findings could provide a new opportunity to reveal PLCs-mediated cell developmental transition during microspore embryogenesis.

## Materials and methods

### Materials

Tobacco plants (*Nicotiana tabacum* L., cv. Petite Havana SR1) were grown under a 16/8 h light/dark cycle at 25°C in green house.

### Phylogenetic analysis

The PLC protein sequences were obtained from NCBI or Uniport (Table S1). For the phylogenetic analysis, the protein sequences were used for multiple sequence alignment with Clustal X 2.0 software. A phylogenetic tree was constructed to analyze the evolutionary relationships of the PLCs by MEGA 5.2 software with the Neighbor-Joining method.

### Microspore isolation and culture

The tobacco buds of 11–12 mm represented unicellular microspores. Anthers were gently dissected from the buds sterilized with 75% ethyl alcohol for 3 min, after three washes with sterilized water, then microspores were released into a dish (706001, NEST) containing B medium^17^ (pH 7.0). Following washing in the same isolation medium twice, microspores were cultured in 2.5 mL B medium in dishes at a density of 5 × 10^4^ grains mL^−1^ and incubated in the dark at 32°C for 5 days. The induced microspores were collected by centrifugation and then transferred into a sugar-containing AT3 medium^18^ (pH 5.6). The microspores were incubated for progression of embryogenesis at 25°C in the dark.

### Quantitative real-time PCR analysis (qRT-PCR)

Total RNA was extracted from in vitro samples using the EZNA® HP Plant RNA Kit (R6837, Omega) according to the manufacturer’s instruction. cDNA was obtained from 2 µg of RNA using the HiScript® II 1st Strand cDNA Synthesis Kit (R211-01, Vazyme). 0.2 μL of resulting first-strand cDNA was used as a PCR template for qRT-PCR. Quantitative PCR analysis were performed using ChamQ SYBR qPCR Master Mix (Q321-02, Vazyme) with a CFX Connect^TM^ Real-Time System (Bio-Rad). The primers used in this study were listed in Table S2. *Ubiquitin-conjugating enzyme 2* (*UBI*) and *Glyceraldehyde-3-phosphate dehydrogenase* (*GAPDH*) were used as the internal references to normalize the relative level of each transcript. Each experiment was repeated three times and each time the experiment included duplicate samples.

### DAPI and FDA staining

Microspores culture were stained with 1 mg mL^−1^ DAPI (D9542, Sigma) for 10 min and analyzed by confocal laser microscopy. To evaluate cell viability, we stained microspores with FDA (fluorescein diacetate, F7378, Sigma). Microspores or microspore embryos were incubated in the solution containing 2 μg mL^−1^ FDA for 10 min at room temperature and washed twice with PBS before observation.^19^

### Neomycin treatment

2.5 μM, 5 μM, 7.5 μM, 10 μM, and 20 μM of neomycin (N6386, Sigma) were added to the microspore culture plates at the time of stress treatment, respectively. Same volume of water was added as a control. After stress treatment, the microspores were transferred into a sugar-containing AT3 medium without neomycin.

### Visualization of lipids

Microspores were collected by centrifugation at 1000 × *g* for 5 min and resuspend by 5% mannitol. Lipids were visualized after the staining with 25 μg mL^−1^ Nile red (RS1294, RYON) dissolved in acetone. Nile red was excited at 546 nm and detected at wavelengths 560–615 nm.

### Confocal microscopy and image analysis

Microspores fluorescence was visualized using a laser scanning confocal microscope (LSM710, Zeiss). The statistics of microspore embryos and cell viability were calculated using ImageJ.

### Quantification and statistical analysis

Mean percentage of the embryos was quantified from random samples of three independent experiments. The fluorescence intensity was calculated as integrated density – (area of selected lipid droplet × mean background fluorescence). Statistical analysis was analyzed using GraphPad Prism 7 software.

## Supplementary Material

Supplemental Material
