# Supplementary material for: Phospholipase C is a novel regulator at the early stages of microspore embryogenesis in Nicotiana tabacum
Source: Plant Signal Behav. 2022 Jul 4;17(1):2094618. doi: 10.1080/15592324.2022.2094618 (PMC9254995; doi:10.1080/15592324.2022.2094618)
Supplement: Supplemental Material [file KPSB_A_2094618_SM6502.docx]

**Supplemental material**

**Phospholipase C is a novel regulator at the early stages of microspore embryogenesis in *Nicotiana tabacum***

Pan Luo^a, #^, Aixi Jiang ^a, #^, Yi Zhou ^a, #^, Mingchun Yang^a^, Xiaotong Zhou^a^, Yong Yang^a^, Jun Yu^b, *^, Xingchun Tang^a, *^

^a^ State Key Laboratory of Biocatalysis and Enzyme Engineering, College of Life Sciences, Hubei University, Wuhan, 430062, China

^b^ Tobacco Research Institute of Hubei Province, Wuhan, 430030, China.

^*^ Correspondence: yujun80324@163.com and tangxingchun@hubu.edu.cn

^#^ These authors contributed equally in this study.

**Contents:**

Figure S1. Evolutionary relationship of PLCs.

Figure S2. Multiple sequence alignment of PLCs.

Table S1. Protein IDs of PLCs used in this study.

Table S2. Premiers used in this study.

**
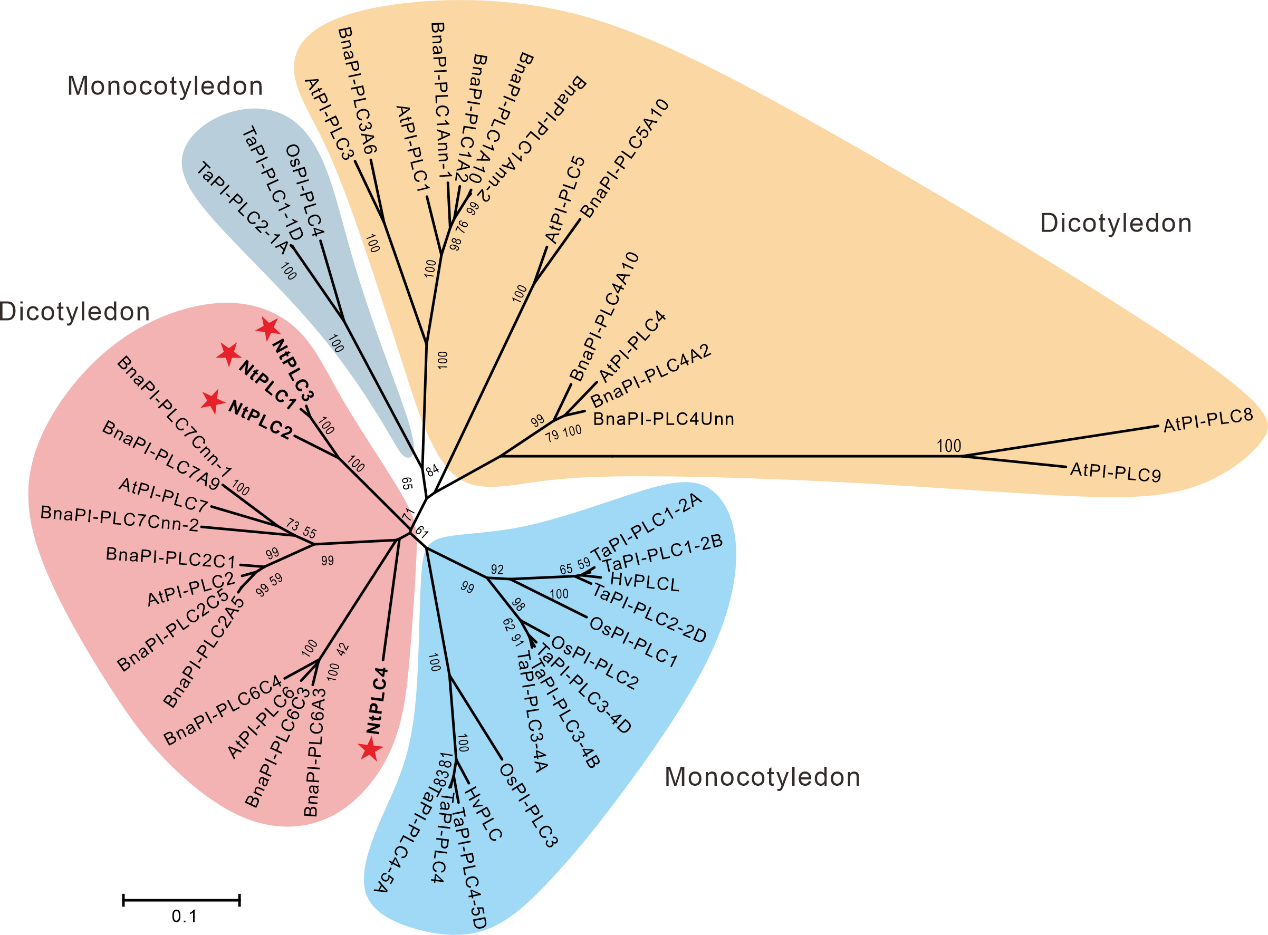
**

**Figure S1. Evolutionary relationship of *PLCs*.**

Phylogenetic analysis of PLC proteins in tobacco (*Nicotiana tabacum*), rice (*Oryza sativa*), wheat (*Triticum aestivum*), rape (*Brassica napus*), barley (*Hordeum vulgare*) and *Arabidopsis thaliana*. The phylogenetic tree was derived with the neighbor-joining method. Numbers indicate bootstrap confidence percentages. Four discrete groups are highlighted in different colors.


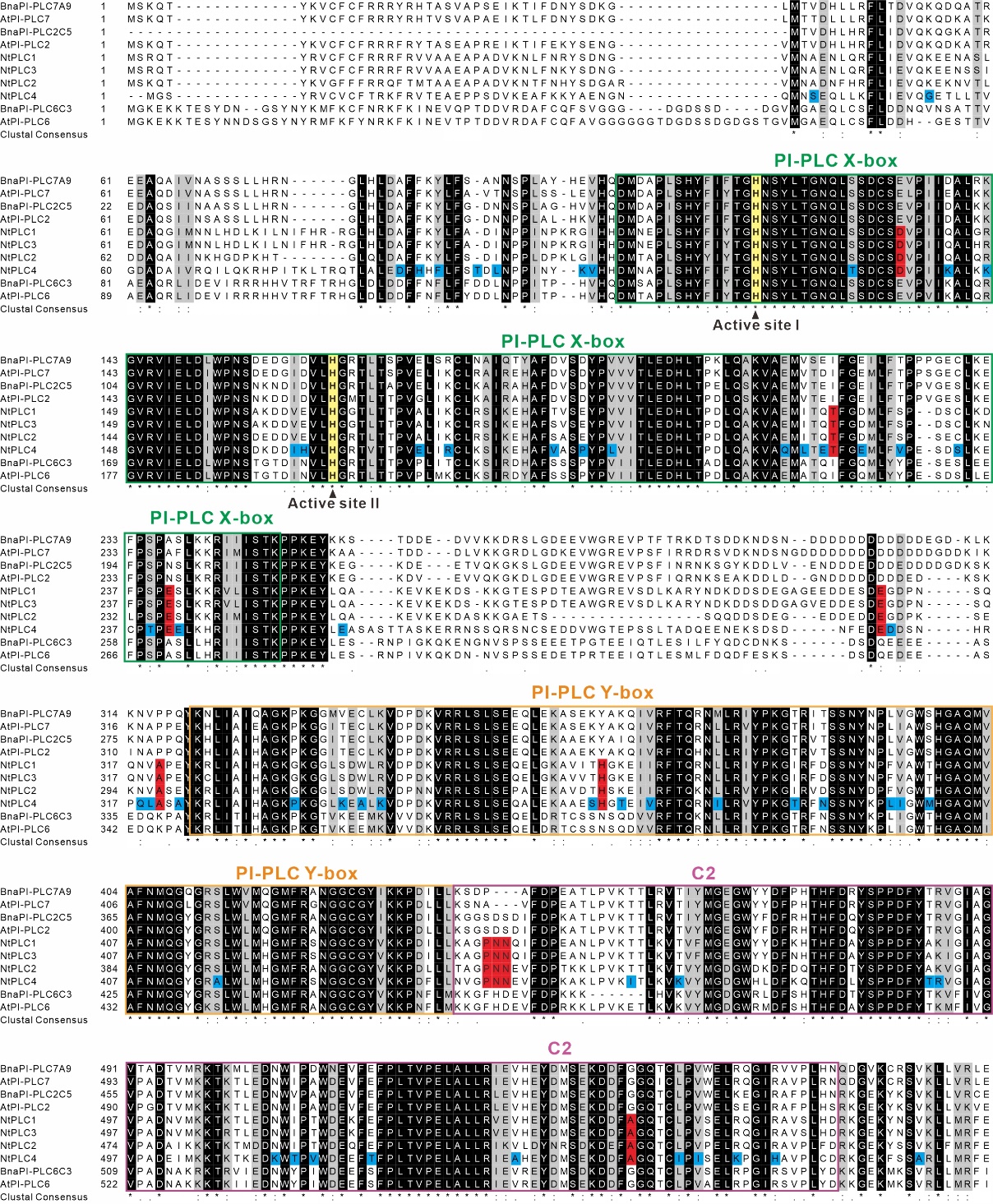


**Figure S2. Multiple sequence alignment of PLCs.**

The green, orange and purple boxes represent PI-PLC-X, PI-PLC-Y, and C2 domains, respectively. Yellow, red and blue shading represent active sites in PI-PLCs, specific amino acid sites in NtPLCs and NtPLC4, respectively.

**Table S1. Protein IDs of PLCs used in this study.**

| **Species** | **Gene name** | **ID** |
| --- | --- | --- |
| *Nicotiana tabacum* | NtPLC1 | AF223351 |
|  | NtPLC2 | AF223573 |
|  | NtPLC3 | EF043044 |
|  | NtPLC4 | EF520286 |
| *Arabidopsis thaliana* | AtPI-PLC1 | AT5G58670.1 |
|  | AtPI-PLC2 | AT3G08510.1 |
|  | AtPI-PLC3 | AT4G38530.1 |
|  | AtPI-PLC4 | AT5G58700.1 |
|  | AtPI-PLC5 | AT5G58690.1 |
|  | AtPI-PLC6 | AT2G40116.1 |
|  | AtPI-PLC7 | AT3G55940.1 |
|  | AtPI-PLC8 | AT3G47290.1 |
|  | AtPI-PLC9 | AT3G47220.1 |
| *Oryza sativa* | OsPI-PLC1 | Os07g49330.1 |
|  | OsPI-PLC2 | Os03g18010.1 |
|  | OsPI-PLC3 | Os12g37560.1 |
|  | OsPI-PLC4 | Os05g03610.1 |
| *Brassica napus* | BnaPI-PLC1A2 | CDY32694 |
|  | BnaPI-PLC1A10 | CDY52120 |
|  | BnaPI-PLC1Ann-1 | CDY61028 |
|  | BnaPI-PLC1Ann-2 | CDY71238 |
|  | BnaPI-PLC1C2 | KAH0896346 |
|  | BnaPI-PLC1C3 | KAH0888808 |
|  | BnaPI-PLC1C9 | KAH0859100 |
|  | BnaPI-PLC2A5 | CDY35983 |
|  | BnaPI-PLC2C1 | CDY22153 |
|  | BnaPI-PLC2C5 | CDY27998 |
|  | BnaPI-PLC3A6 | CDY70829 |
|  | BnaPI-PLC4A2 | CDY32691 |
|  | BnaPI-PLC4A10 | CDY52122 |
|  | BnaPI-PLC4C9 | KAH0859103 |
|  | BnaPI-PLC4Unn | CDY71544 |
|  | BnaPI-PLC5A10 | CDY52121 |
|  | BnaPI-PLC5C9 | KAH0859102 |
|  | BnaPI-PLC6A3 | CDY07623 |
|  | BnaPI-PLC6A4 | KAH0930332 |
|  | BnaPI-PLC6A5 | KAH0925001 |
|  | BnaPI-PLC6C3 | CDY60344 |
|  | BnaPI-PLC6C4 | CDY20520 |
|  | BnaPI-PLC7A4 | KAH0928756 |
|  | BnaPI-PLC7A9 | CDY27254 |
|  | BnaPI-PLC7C8 | KAH0864458 |
|  | BnaPI-PLC7Cnn-1 | CDY70294 |
|  | BnaPI-PLC7Cnn-2 | CDY47356 |
| *Triticum aestivum* | TaPI-PLC4-5B | A0A3B6LIH5 |
|  | TaPI-PLC3-4A | A0A3B6HQB3 |
|  | TaPI-PLC1-2D | A0A3B6D6L9 |
|  | TaPI-PLC2-1A | A0A3B5XW63 |
|  | TaPI-PLC4-5A | A0A3B6KDX2 |
|  | TaPI-PLC3-4D | A0A3B6JJX5 |
|  | TaPI-PLC1-2A | A0A3B6AR20 |
|  | TaPI-PLC3-4B | A0A3B6IRU2 |
|  | TaPI-PLC4-5D | A0A3B6MPW1 |
|  | TaPI-PLC1-2B | XP_044321915.1 |
|  | TaPI- PLC1-1D | XP_044448189.1 |
| *Hordeum vulgare* | HvPLC | AK360343.1 |
|  | HvPLCL | AK370341.1 |

**Table S2. Premiers used in this study.**

| Primer | Sequence | |
| --- | --- | --- |
| NtPLC1-F | GATGCATACACGCCTCCAGA |  |
| NtPLC1-R | TGGGGTATCCATATCCTCCAGT | |
| NtPLC2-F | GGAAAAAGATGCCTCAAAGAAAGGA | |
| NtPLC2-R | GGTCAACCCTCAGCCAATCA | |
| NtPLC3-F | AGGATGATTCTGATGACGGAGC | |
| NtPLC3-R | CAGGATCAACCCTCAGCCAA | |
| NtPLC4-F | GGGGAGTTATAGAGTATGTGTGTGT | |
| NtPLC4-R | TTCATTTGGTTCCCATTTTCAGCA | |
| UBI-F | GCGGTGGTATGCAGATTTTC | |
| UBI-R | TCCTGCAAAGATCAGCCTCT | |
| GAPDH-F | AGGCTGGAGAAAGAAGCTACCTA | |
| GAPDH-R | AGTCTGTGGACACCACATCATCT | |
